# Dietary Food-Group Diversity Is Associated with Lower Frailty Severity in Older Adults Undergoing Geriatric Outpatient Assessment

**DOI:** 10.3390/foods15152638

**Published:** 2026-07-28

**Authors:** Simone Perna, Gaetan Claude Barrile, Giuseppe Mazzola, Mariangela Rondanelli

**Affiliations:** 1 Department of Food, Environmental and Nutritional Sciences, Division of Human Nutrition, University of Milan, 27100 Milan, Italy; 2 Endocrinology and Nutrition Unit, Azienda di Servizi alla Persona “Istituto Santa Margherita”, University of Pavia, 27100 Pavia, Italygiuseppe.mazzola02@universitadipavia.it (G.M.); 3 Department of Public Health, Experimental and Forensic Medicine, University of Pavia, 27100 Pavia, Italy

**Keywords:** DEXA, dietary diversity, frailty, Edmonton Frail Scale, Mini Nutritional Assessment, geriatrics, cross-sectional study

## Abstract

Background: Dietary diversity has been proposed as a modifiable determinant of healthy ageing, but its association with objectively assessed frailty in geriatric outpatients remains poorly characterised. We examined the cross-sectional association between a four-item Dietary Diversity Score (DDS) and frailty severity, and whether nutritional status statistically mediates this association. Methods: Cross-sectional study of 317 older adults (median age 82 years, 73.8% female) attending a geriatric outpatient unit in Northern Italy. Consumption of four food groups (dairy, legumes/eggs, meat/fish/poultry, fruits/vegetables) was summed into a DDS (0–4). Frailty was measured with the Edmonton Frail Scale (EFS). Mediation by the Mini Nutritional Assessment (MNA) was tested with bootstrapping; individual food-group associations across eight outcomes were corrected for multiple testing (Benjamini–Hochberg). Results: Each additional food group was associated with a 0.61-point-lower EFS score after adjusting for age, sex and BMI (β = −0.608; *p* = 0.001; adjusted R^2^ ≈ 0.04–0.07) and 36% lower odds of higher frailty severity (OR = 0.64; *p* < 0.001). MNA statistically explained 86.2% of the DDS-EFS association (bootstrap 95%CI excluding zero), and the significant mediation paths remained robust after FDR correction (q ≤ 0.0015). Legumes/eggs showed the strongest food-specific association with EFS and MNA after correction (q ≤ 0.002); fruit/vegetable associations with inflammatory and nutritional biomarkers did not survive correction and are exploratory. Conclusions: Dietary diversity is associated with lower frailty severity in geriatric outpatients, largely explained by nutritional status; the modest explanatory power reflects frailty’s multifactorial aetiology, and prospective studies are needed to test causality.

## 1. Introduction

Frailty is a multidimensional geriatric syndrome characterised by reduced physiological reserve and increased vulnerability to adverse health outcomes, including falls, hospitalisation, functional decline, and mortality [1,2]. Its prevalence ranges from approximately 10–20% in community-dwelling older adults to over 50% in hospitalised patients, placing a substantial burden on health systems worldwide [3]. Identifying modifiable determinants of frailty is therefore a global priority for preventive geriatrics.

Among potentially modifiable factors, nutrition occupies a central position. Mechanistically, adequate dietary intake sustains skeletal muscle protein synthesis, bone mineralisation, mitochondrial integrity, and immune competence—all biological processes implicated in the frailty phenotype [4,5]. Epidemiological evidence consistently links adherence to high-quality dietary patterns [6] and adequate protein intake with lower frailty risk; recent meta-analytic evidence further suggests that higher protein consumption—particularly animal-derived protein, in observational data—is associated with lower incident frailty risk [7]. Consistent with this, a large cross-sectional analysis of the U.S. NHANES cohort spanning a wide adult age range reported that higher diet-quality scores were associated with lower frailty and lower mortality risk [8]. That study evaluated a composite, multi-component diet-quality index in a large community-dwelling US population spanning young to older adults, and did not examine dietary diversity specifically, nor test whether the association with frailty is statistically mediated by nutritional status. The present study addresses this distinct question in a geriatric clinical outpatient population using a simple four-item Dietary Diversity Score and a formal Baron–Kenny mediation analysis, which to our knowledge has not previously been reported. Conversely, malnutrition, which is highly prevalent in older adults requiring geriatric care, is a powerful independent predictor of frailty and functional decline [9,10].

Quantitative dietary assessments such as 24 h dietary recall interviews and food frequency questionnaires are cumbersome to administer in frail older populations and require specialised personnel and processing resources. Dietary diversity, operationalised as the number of distinct food groups consumed, offers a practical, low-burden proxy for overall diet quality [11]. The Dietary Diversity Score (DDS) has been validated against micronutrient adequacy across multiple populations [12] and has been associated with lower cognitive decline risk in community-dwelling older adults in Japan [13]. More recent cohort studies have linked baseline DDS and longitudinal changes in DDS with subsequent frailty risk [14,15], and a 2024 systematic review and meta-analysis reported that low dietary diversity was associated with higher frailty risk in older adults [16]. Nevertheless, studies examining DDS in geriatric outpatient cohorts assessed with objective instruments combining body composition measurement (dual-energy X-ray absorptiometry, DEXA), standardised frailty scales, and nutritional assessment are still relatively few. Of note, the Edmonton Frail Scale and allied geriatric instruments (MMSE, MNA, ADL/IADL, handgrip strength, and DEXA-derived body composition indices) were previously characterised in a hospitalised Italian cohort drawn from the same care network [17]; while that work validated the multidimensional performance of the frailty scale itself, the present study addresses a distinct question, namely the association of dietary food-group diversity with frailty severity and its statistical mediation by nutritional status in a geriatric outpatient sample.

An additional question concerns whether nutritional status statistically accounts for the DDS–frailty relationship. One plausible, non-causal explanation is that a diverse diet may be a marker of better protein–energy intake, appetite, autonomy in food choice, and social or functional capacity; these factors may in turn correlate with muscle wasting, immune competence, and functional decline. The Mini Nutritional Assessment (MNA), one of the most validated nutritional screening instruments in older adults [9], captures this dimension. Whether MNA statistically mediates the dietary diversity–frailty association has not, to our knowledge, been formally evaluated in an older Italian geriatric outpatient cohort.

This study therefore aimed to (i) characterise the cross-sectional dose–response relationship between a four-item DDS and frailty severity (EFS) in a large cohort of geriatric outpatients; (ii) examine the associations of individual food groups with frailty and related clinical outcomes; (iii) formally test whether nutritional status (MNA) statistically mediates the DDS–EFS association; and (iv) assess sex differences in line with SAGER guidelines [18].

## 2. Materials and Methods

### 2.1. Study Design and Setting

This cross-sectional observational study is reported in accordance with the STROBE checklist [19]. Data were obtained from a prospective clinical registry of consecutive patients attending the geriatric outpatient clinic of the Azienda di Servizi alla Persona “Istituto Santa Margherita”, Pavia, Italy, between January 2018 and February 2024. The registry was established to prospectively capture all patients undergoing comprehensive geriatric assessment (CGA) for multimorbidity, functional decline, suspected malnutrition, or sarcopenia.

### 2.2. Participants

**Figure 1:**
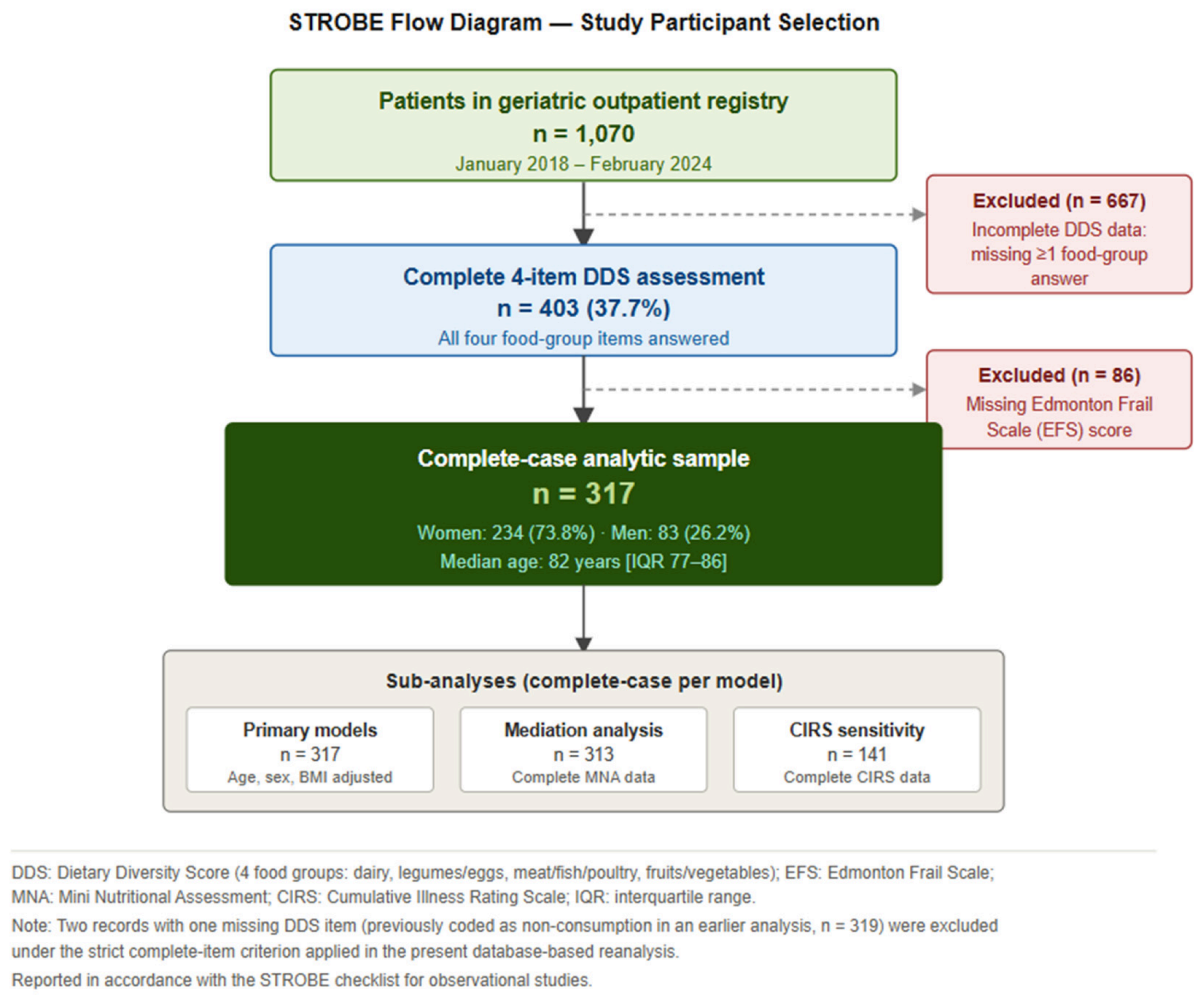
Flow chart of the study.

Inclusion criteria: age ≥ 65 years; availability of complete answers for all four dietary group items and EFS score at the index assessment. Exclusion criteria: acute illness requiring inpatient hospitalisation at assessment; terminal illness with life expectancy < 3 months. The full cohort comprised 1070 patients; 403 had complete answers for all four DDS food-group items, and 317 of these also had a complete EFS assessment and formed the updated complete-case analytic sample (see STROBE flow diagram, Figure 1). Two records that had previously been conservatively coded as non-consumption for one missing dietary item were excluded in the present database-based reanalysis; sensitivity analyses using the previous coding were materially unchanged.

### 2.3. Dietary Diversity Score (DDS)

At each assessment, a trained nurse or dietitian administered a brief structured dietary inquiry, recording whether the patient had consumed each of the following four food groups in the preceding week: (i) dairy products and milk; (ii) legumes and eggs; (iii) meat, fish, and poultry; and (iv) fruits and vegetables. Responses were binary (1 = yes, 0 = no). The DDS was computed as the integer sum (range 0–4). Participants were included only if an answer for each of the four items was recorded. For analyses, DDS was treated as continuous (primary) and as a three-level categorical variable: low (0–2, *n* = 50), medium (3, *n* = 101), and high (4, *n* = 166).

### 2.4. Primary Outcome: Edmonton Frail Scale (EFS)

Frailty was assessed by the Edmonton Frail Scale (EFS), a validated nine-domain, 11-item instrument (cognition, general health, functional independence, social support, medications, mood, continence, nutrition, functional performance) with scores ranging from 0 (not frail) to 17 (severely frail) [20]. Categorical EFS cut-points were applied for secondary analyses: non-frail 0–4, vulnerable 5–6, mild 7–8, moderate 9–10, severe ≥ 11.

### 2.5. Nutritional Status: Mini Nutritional Assessment (MNA)

The Mini Nutritional Assessment Full Form (MNA-FF; 18 items, range 0–30) was administered as part of the CGA. Scores ≥ 24 indicate adequate nutritional status; 17–23.5 indicate risk of malnutrition; <17 indicate malnutrition [9].

### 2.6. Covariates

Covariates were selected a priori based on clinical plausibility: age (years, continuous); sex (binary); BMI (kg/m^2^, continuous); Cumulative Illness Rating Scale (CIRS) severity score; and polypharmacy (number of medications). The CIRS is a clinician-rated index of chronic medical illness burden across organ systems; in this registry, CIRS items were completed during the CGA from medical history, diagnoses, medication lists, and clinical documentation, and the severity score was used as an aggregate measure of comorbidity burden [21]. Given 55.5% missingness for CIRS, the primary model excluded CIRS (Model 2, *n* = 317), while a sensitivity model additionally adjusted for CIRS (Model 3, *n* = 141).

### 2.7. Additional Assessments

Body composition was measured by dual-energy X-ray absorptiometry (DEXA; Hologic Discovery A or similar) and bioimpedance analysis (BIA; AKERN BIA 101 or similar). Sarcopenia-related outcomes included Skeletal Muscle Index (SMI = appendicular lean mass/height^2^, kg/m^2^) and handgrip dynamometry (Jamar, dominant hand). Bone health was assessed by lumbar spine T-score (DEXA) and 10-year fracture probability (FRAX, WHO model). Biochemical parameters included serum albumin, C-reactive protein (CRP), 25-hydroxyvitamin D, and haematological indices from routine venepuncture. Functional capacity was evaluated by ADL (Katz index), IADL (Lawton index), and MMSE; for ADL and IADL, higher scores indicate better function.

### 2.8. Statistical Analysis

Continuous variables are presented as median [IQR]; categorical as count (%). Group comparisons across DDS tertiles used the Kruskal–Wallis test (continuous) and chi-square test (categorical).

Individual food-group analyses: For each of the four food groups, unadjusted associations with all clinical outcomes were tested using Mann–Whitney U test. Adjusted associations were quantified using multivariable linear regression (β coefficient, 95%CI) with the covariates age, sex, and BMI. Primary analysis: DDS and EFS. The association between DDS (continuous) and EFS (continuous) was modelled across four nested linear regression models: crude (Model 1); adjusted for age, sex, BMI (Model 2, primary); additionally adjusted for CIRS and polypharmacy (Model 3, sensitivity, *n* = 141); and additionally including MNA (Model 4, statistical mediation test, *n* = 313). An ordered logistic regression model (Model 5) was fitted with EFS in five ordinal categories.

Regression diagnostics were assessed by residual-versus-fitted plots, Q-Q plots, leverage/influence checks, variance inflation factors for multicollinearity, Breusch–Pagan testing for heteroscedasticity, Ramsey RESET testing for model specification, and threshold-specific binary logistic models as a practical check of the ordered-logit proportional-odds assumption.

Mediation analysis: The Baron–Kenny causal steps framework [22] was applied to the DDS-MNA-EFS statistical mediation analysis. Three equations were estimated: DDS → EFS (path c); DDS → MNA (path a); DDS + MNA → EFS (paths b and c′). The indirect association (a × b) and its 95%CI were estimated by non-parametric bootstrapping (2000 resamples). Significant statistical mediation was inferred when the bootstrap CI excluded zero. Proportion mediated = |a × b/c| × 100. Because exposure, mediator, and outcome were assessed cross-sectionally, mediation estimates were interpreted as statistical mediation rather than evidence of temporal or causal mediation.

Sex-stratified analyses (SAGER guidelines):

All primary models were replicated separately in women and men [18]. Sex modification was tested with a DDS × sex interaction term in the pooled model.

Sensitivity analyses: DDS was operationalised as binary (DDS = 4 vs. <4). Frailty was dichotomised as EFS ≥ 7 and modelled with logistic regression. All models were refitted after excluding participants aged <70 years (*n* = 17) to verify results in the core geriatric age range.

Missing data: Patients with complete dietary data were compared with registry patients who lacked complete dietary data on all key clinical variables; patients without complete dietary data were not included in the analytic regression models.

Regression models used complete-case analysis, and sample sizes are reported explicitly for each model. Analyses were performed in Python 3.12 using SciPy 1.11, StatsModels 0.14, and the custom Baron–Kenny bootstrap procedure. Two-sided *p* < 0.05 was considered significant; multiple comparisons in Table 3 are exploratory and should be interpreted with caution. To address multiplicity across the individual food-group models, the adjusted *p*-values for these analyses were additionally controlled using the Benjamini–Hochberg false-discovery-rate (FDR) procedure, and FDR-adjusted q-values are reported alongside nominal *p*-values (Table 3). The same FDR procedure was additionally applied to the four inferential path tests of the mediation model (Table 4).

### 2.9. Ethics

The study was conducted in accordance with the Declaration of Helsinki. All patients provided written informed consent for data use for research. Ethics approval was obtained from the Comitato Etico Territoriale Lombardia (CET Lombardia) (approval number 1219/12062024, 12 June 2024). Data were fully anonymised prior to analysis.

## 3. Results

### 3.1. Subject Cohort and Missing Data Analysis

Of 1070 patients in the registry, 403 (37.7%) had complete answers for all four dietary group items; 317 of these also had a complete EFS assessment and formed the updated complete-case analytic sample. Two records with one missing food-group answer and complete EFS data were excluded from the present reanalysis rather than being conservatively coded as non-consumption. Patients excluded from the analytic sample were statistically comparable to included patients on age (median 81 vs. 82 years), EFS (8.0 vs. 8.0), MNA (18.0 vs. 18.0), BMI, sex, MMSE, SMI, and handgrip strength (all *p* > 0.05). Excluded patients had a higher number of medications (median 10 vs. 8; *p* < 0.001) and a slightly higher diagnostic burden by rank distribution (median 6 vs. 6; *p* = 0.030), indicating that selection bias cannot be fully excluded.

### 3.2. Descriptive Characteristics

The analytic sample (*n* = 317) had a median age of 82 years (IQR 77–86), was 73.8% female, and had a median EFS of 8.0 (IQR 6–10), spanning the mild-to-moderate frailty range. Polypharmacy was prevalent (median 8 medications [IQR 6–10]). The majority of patients consumed all four food groups (DDS = 4; 52.4%); 31.9% consumed three groups, and 15.8% consumed two or fewer. Additional cohort characteristics confirmed substantial clinical complexity: cardiovascular diseases were present in 81.5%, osteomuscular/connective-tissue diseases in 51.1%, digestive diseases in 23.9%, diabetes in 19.5%, previous fractures in 19.1%, tumours in 10.2%, and current smoking in 6.0% of patients with available data. Baseline characteristics by DDS group are reported in Table 1.

The three DDS groups were broadly comparable across most demographic and clinical severity indicators (Table 1), including sex, BMI, medications, comorbidities, CIRS severity, albumin, CRP, and vitamin D. Age differed modestly across DDS groups (*p* = 0.045), and age was therefore retained as an a priori covariate in all adjusted models. EFS and MNA differed significantly across groups (*p* = 0.008 and *p* < 0.001, respectively), supporting the clinical relevance of the DDS gradient.

### 3.3. Dose–Response: DDS and Frailty Severity

**Figure 2:**
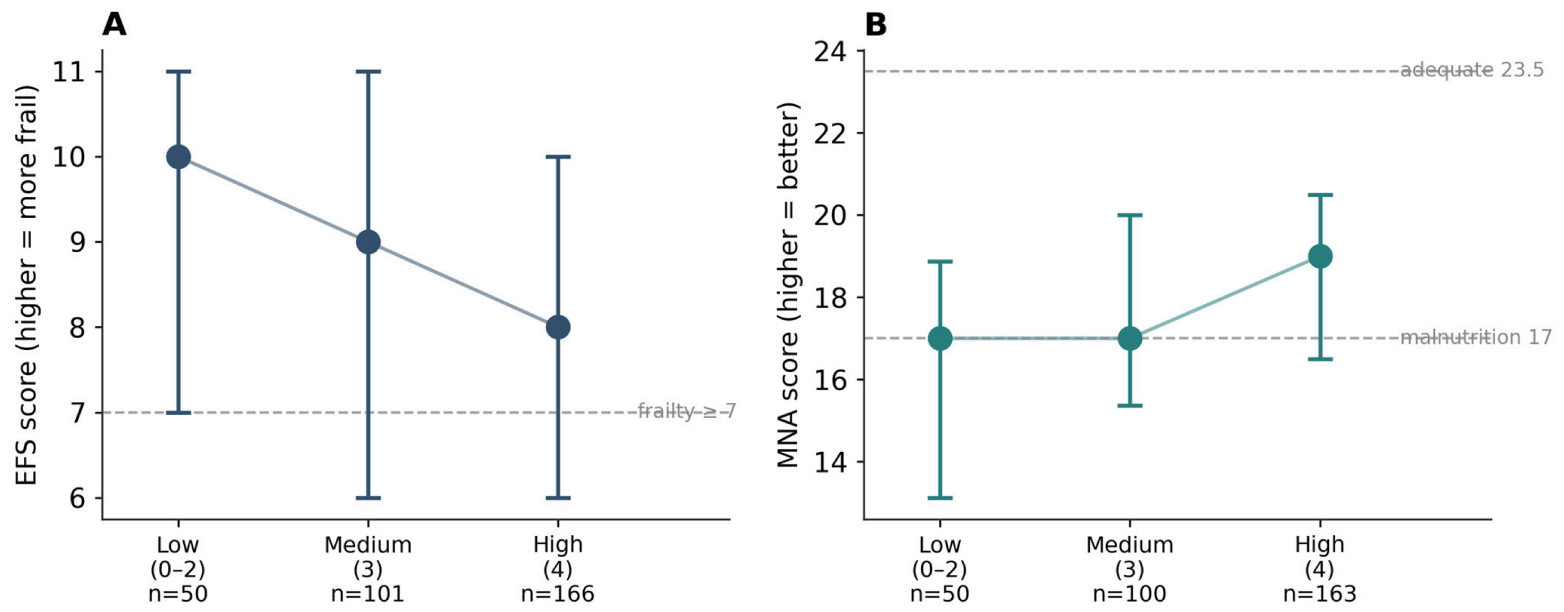
Dose–response relationship between Dietary Diversity Score (DDS) and geriatric outcomes. Panel (**A**): EFS score (median [IQR]) by DDS level; dashed line = frailty threshold (EFS ≥ 7). Panel (**B**): MNA score (median [IQR]) by DDS level; dashed lines = malnutrition risk (17) and adequate nutrition (23.5) thresholds. n per group shown on x-axis. Statistical comparisons: Kruskal–Wallis overall test. Post hoc low-vs.-high contrasts and exact *p*-values are reported in Section 3.3 and shown within the panels; the redundant in-caption values have been removed to improve readability.

EFS decreased monotonically with increasing DDS: median EFS was 11.0 at DDS = 0, 9.0 at DDS = 2–3, and 8.0 at DDS = 4 (Kruskal–Wallis H = 9.59, *p* = 0.008). Severe frailty (EFS ≥ 11) was observed in 38.0% of the low-DDS group, 29.7% of the medium-DDS group, and 15.1% of the high-DDS group. Conversely, MNA showed a dose-dependent increase with DDS, from median 10.8 (DDS = 0) to 19.0 (DDS = 4; Spearman rho = +0.281, *p* < 0.001). Post hoc pairwise comparisons were performed between the predefined low-DDS group (DDS 0–2) and high-DDS group (DDS = 4), confirming significant differences for both EFS (Mann–Whitney *p* = 0.003) and MNA (*p* < 0.001) (Figure 2).

### 3.4. Multivariable Regression: DDS and EFS

Table 2 summarises all regression models. In the crude model (Model 1), each unit increase in DDS was associated with −0.660 EFS points (95%CI −1.024, −0.296; *p* < 0.001). After adjustment for age, sex, and BMI (Model 2, primary model), the coefficient was essentially unchanged (β = −0.608; 95%CI −0.976, −0.239; *p* = 0.001; *n* = 317), confirming independence from demographic confounders. The sensitivity model including CIRS and polypharmacy (Model 3) yielded a similar coefficient (β = −0.773; 95%CI −1.290, −0.256; *p* = 0.004; *n* = 141), with the reduction in sample size attributable to CIRS missingness. Ordered logistic regression (Model 5) estimated each additional food group to reduce the odds of being in a higher frailty category by 36% (OR 0.64; 95%CI 0.50–0.82; *p* < 0.001). Although the DDS–EFS association was statistically robust, the demographically adjusted models explained only a small proportion of the variance in frailty severity (adjusted R^2^ ≈ 0.04–0.07; Table 2). This is expected given the multifactorial aetiology of frailty and indicates that dietary diversity should be regarded as one modest correlate among many, rather than a dominant determinant of frailty.

### 3.5. Individual Food-Group Associations (Table 3)

**Figure 3:**
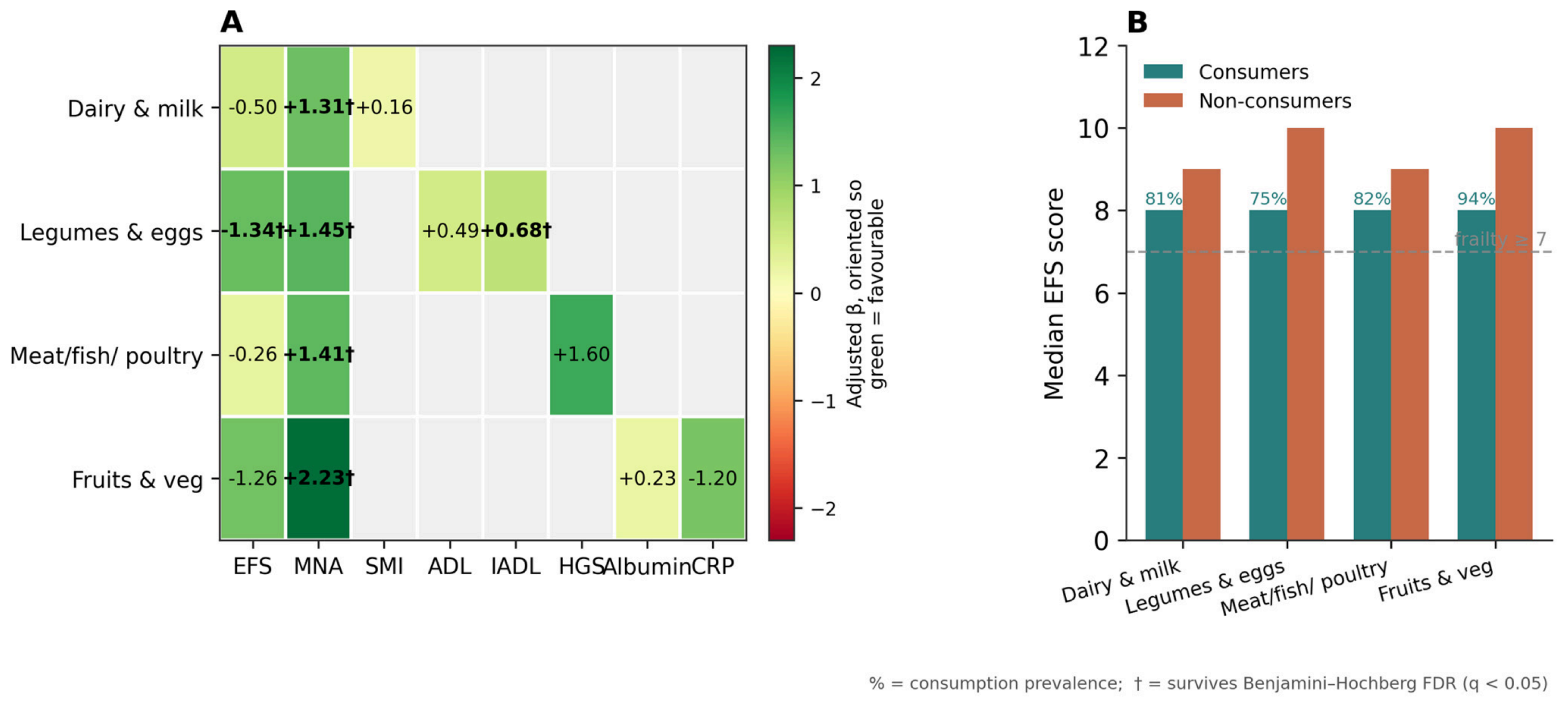
Individual food-group associations with frailty and clinical outcomes. Panel (**A**): heatmap of adjusted β coefficients (OLS, adjusted for age, sex, BMI) for each food group–outcome combination; green = favourable direction, red = unfavourable. Panel (**B**): EFS medians for consumers vs. non-consumers by food group; error bars = IQR; percentage labels = consumption prevalence. MW: Mann–Whitney.

The individual food-group analysis (Table 3) revealed heterogeneous patterns. All four food groups were independently associated with MNA in adjusted models (β range +1.309 to +2.225; *p* ≤ 0.003), confirming the primacy of the nutritional pathway (Figure 3).

**Table 3:** Individual food-group associations with clinical outcomes: unadjusted (Mann–Whitney) and adjusted (OLS) analyses.

Food Group	Outcome	Median (Yes)	Median (No)	n Yes/ n No	*p* (MW)	**β** adj	*p* adj
Dairy & milk	MNA score	18.5	17.0	252/61	0.002 **	+1.309	0.003 **
Dairy & milk	SMI (kg/m^2^)	6.64	6.21	254/60	0.009 **	+0.163	0.240 ns
Dairy & milk	EFS score	8.0	9.0	256/61	0.316 ns	−0.503	0.222 ns
Legumes & eggs	EFS score	8.0	10.0	239/78	<0.001 ***	−1.339	<0.001 ***
Legumes & eggs	MNA score	18.5	16.5	236/77	<0.001 ***	+1.451	<0.001 ***
Legumes & eggs	IADL score	1.0	0.0	210/69	0.004 **	+0.678	0.016 *
Legumes & eggs	ADL score	3.0	3.0	213/71	0.046 *	+0.489	0.048 *
Meat, fish & poultry	MNA score	18.5	17.0	257/56	0.008 **	+1.412	0.002 **
Meat, fish & poultry	Handgrip DX (kg)	16.0	15.0	198/49	0.017 *	+1.600	0.081 ns
Meat, fish & poultry	EFS score	8.0	9.0	261/56	0.390 ns	−0.262	0.538 ns
Fruits & vegetables	EFS score	8.0	10.0	297/20	0.027 *	−1.262	0.058 ns
Fruits & vegetables	MNA score	18.5	16.3	293/20	0.009 **	+2.225	0.002 **
Fruits & vegetables	Albumin (g/dL)	3.69	3.48	282/20	0.042 *	+0.226	0.043 *
Fruits & vegetables	CRP (mg/L)	0.32	0.69	286/18	0.086 ns	−1.204	0.035 *

β adj: adjusted β coefficient from multivariable OLS (covariates: age, sex, BMI). MW: Mann–Whitney U test. * *p* < 0.05; ** *p* < 0.01; *** *p* < 0.001; ns: not significant.

Legumes and eggs showed the strongest and most consistent multi-domain associations: significantly lower EFS (β = −1.339; *p* < 0.001) and higher MNA (β = +1.451; *p* < 0.001) in adjusted models, as well as better ADL (*p* = 0.048) and IADL (*p* = 0.016) functional scores. This food group was the only one independently associated with frailty after covariate adjustment.

Fruits and vegetables, despite the highest consumption rate (93.7%), were associated with lower EFS in unadjusted analysis (*p* = 0.027), and significantly better albumin (β = +0.226; *p* = 0.043) and lower CRP (β = −1.204; *p* = 0.035) in adjusted analyses, suggesting a possible anti-inflammatory pathway. The adjusted EFS association did not retain statistical significance (β = −1.262; *p* = 0.058), likely reflecting reduced statistical power due to the small non-consumer group (*n* = 20). Dairy products were primarily associated with nutritional status (β = +1.309 for MNA; *p* = 0.003) without an independent adjusted association with EFS. Meat, fish, and poultry also showed a robust MNA association (β = +1.412; *p* = 0.002) and a borderline handgrip association (β = +1.600; *p* = 0.081).

After Benjamini–Hochberg FDR correction across the 14 adjusted food-group tests, the following associations remained significant at q < 0.05: legumes/eggs–EFS (q = 0.002), legumes/eggs–MNA (q = 0.002), legumes/eggs–IADL (q = 0.037), dairy–MNA (q = 0.009), meat/fish/poultry–MNA (q = 0.007), and fruits/vegetables–MNA (q = 0.007). The fruits/vegetables–albumin (q = 0.075), fruits/vegetables–CRP (q = 0.071), legumes/eggs–ADL (q = 0.075), and fruits/vegetables–EFS (q = 0.081) associations did not survive FDR correction and are therefore considered exploratory.

### 3.6. Mediation by Nutritional Status (MNA)

Formal statistical mediation results are shown in Table 4. DDS was associated with EFS in the total-effect model (path c: β = −0.620; 95%CI −0.990, −0.249; *p* = 0.001) and was also associated with MNA (path a: β = +1.119; 95%CI +0.732, +1.507; *p* < 0.001). MNA in turn was associated with EFS after adjustment (path b: β = −0.477; 95%CI −0.570, −0.384; *p* < 0.001). When both DDS and MNA were entered simultaneously, the direct DDS-EFS association became non-significant (path c′: β = −0.085; *p* = 0.620). The bootstrapped indirect association was −0.534 (95%CI −0.770, −0.323), with a CI entirely excluding zero. The proportion of the total association statistically explained by MNA was 86.2%. Because the data are cross-sectional, these estimates should be interpreted as statistical mediation, not proof of temporal or causal mediation.

**Table 4:** Statistical mediation analysis: DDS → MNA → EFS (Baron–Kenny, *n* = 313).

Path	Predictor → Outcome	β Coefficient	95%CI	*p*-Value	Interpretation
c (total)	DDS → EFS	−0.620	[−0.990, −0.249]	0.001 **	Total association
a	DDS → MNA	+1.119	[+0.732, +1.507]	<0.001 ***	Path to mediator
b	MNA → EFS	−0.477	[−0.570, −0.384]	<0.001 ***	Mediator → outcome
c′ (direct)	DDS → EFS	−0.085	[−0.424, +0.253]	0.620 ns	After controlling MNA
a × b (indirect)	DDS → MNA → EFS	−0.534	[−0.770, −0.323]	Bootstrap 95%CI	86.2% proportion mediated

All paths adjusted for age, sex, BMI. Indirect association (path a × b) estimated by non-parametric bootstrap (2000 resamples). Proportion statistically mediated = |indirect/total| × 100 = 86.2%. DDS: Dietary Diversity Score; MNA: Mini Nutritional Assessment; EFS: Edmonton Frail Scale. ** *p* < 0.01; *** *p* < 0.001; ns: non-significant.

Because this table reports four inferential path tests, we additionally applied Benjamini–Hochberg FDR correction across the four *p*-values in paths c, a, b and c′ (q < 0.05 threshold). Paths c (q = 0.0015), a (q < 0.001) and b (q < 0.001) remain significant by a wide margin after correction; the direct path c′ remains non-significant (q = 0.620). This pattern is consistent with strong statistical attenuation of the DDS-EFS association after inclusion of MNA, but not with proof of causal mediation. No initially significant path in this table loses significance under correction.

### 3.7. Sex-Stratified Analyses

In women (*n* = 234), the DDS-EFS association was significant (β = −0.672; 95%CI −1.097, −0.247; *p* = 0.002). In men (*n* = 83), the direction was consistent but non-significant (β = −0.253; 95%CI −1.026, +0.520; *p* = 0.517), most plausibly due to reduced statistical power. The interaction term DDS × sex was non-significant (β = +0.214; *p* = 0.631), indicating no evidence of sex modification. The DDS-MNA association was significant in both women (Spearman rho = +0.285; *p* < 0.001) and men (rho = +0.277; *p* = 0.012).

**Figure 4:**
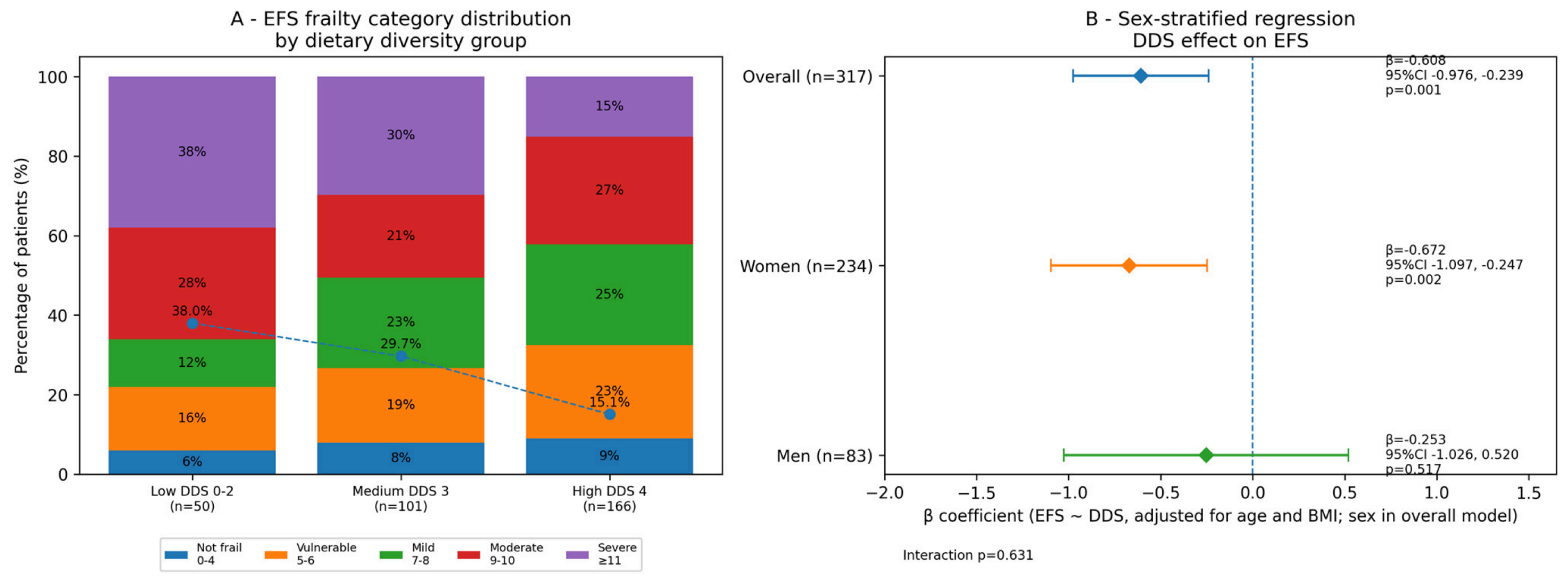
Frailty category distribution and sex-stratified analysis after complete-case database reanalysis. Panel (**A**): stacked bar chart showing EFS frailty category distribution (%) by DDS group (low, medium, high); dashed line traces the prevalence of severe frailty. Panel (**B**): forest plot of adjusted β coefficients (DDS effect on EFS) in the overall sample, women, and men; diamonds = β estimate, horizontal bars = 95%CI; dashed vertical line = null effect. Interaction *p* = 0.631 (not significant).

The distribution of EFS frailty categories by DDS group and the sex-stratified forest plot are shown in Figure 4. The progressive shift from severe frailty (38.0% to 15.1%) toward vulnerability and mild frailty with increasing DDS is visually apparent in the stacked bar chart.

### 3.8. Sensitivity Analyses

When DDS was operationalised as binary (DDS = 4 vs. DDS < 4), consuming all four food groups remained associated with −0.793 lower EFS points (95%CI −1.432, −0.155; *p* = 0.015). Logistic regression for binary frailty (EFS ≥ 7) yielded OR 0.80 (95%CI 0.58–1.09; *p* = 0.160), non-significant, likely reflecting power loss from binary dichotomisation and the high base rate of frailty. Restriction to patients aged ≥ 70 years (*n* = 295) did not materially change the primary coefficient (β = −0.592; 95%CI −0.968, −0.215; *p* = 0.002). As an additional sensitivity analysis addressing CIRS missingness, adjustment for number of diagnoses and number of medications in the larger available sample (*n* = 312) again produced a similar DDS-EFS association (β = −0.612; 95%CI −0.972, −0.252; *p* < 0.001). Linear-model diagnostics were acceptable: residual normality (Shapiro *p* = 0.380), homoscedasticity (Breusch–Pagan *p* = 0.451), model specification (Ramsey RESET *p* = 0.791), and multicollinearity (all variance inflation factors < 1.05) did not indicate major violations. HC3 robust standard errors confirmed the main DDS association (β = −0.608; 95%CI −0.966, −0.249; *p* < 0.001).

## 4. Discussion

### 4.1. Summary of Findings

In this cross-sectional study of 317 older adults undergoing comprehensive geriatric assessment, greater dietary food-group diversity, quantified by a simple four-item score, was associated with lower frailty severity after adjustment for key confounders. The relationship followed a dose–response gradient, with severe frailty declining from 38.0% to 15.1% across DDS groups. Formal statistical mediation analysis indicated that much of the DDS-EFS association was explained by nutritional status, as assessed by MNA, although the cross-sectional design precludes temporal or causal interpretation. Among individual food groups, legumes and eggs showed the most consistent multi-domain associations, including lower frailty and better functional status. Together, these findings support the integration of brief dietary diversity screening, feasible within any CGA, as an early indicator of nutritional and functional risk.

### 4.2. Dietary Diversity and Frailty: Contextualisation

Our findings extend the growing literature on diet quality and frailty. The meta-analysis by Kojima et al. [6] established that adherence to the Mediterranean diet is associated with lower incident frailty risk; Sandoval-Insausti et al. [23] showed that macronutrient distribution, particularly protein adequacy, predicts frailty incidence in prospective cohorts. More recent evidence has extended this literature to dietary diversity specifically: Duan et al. observed prospective DDS–frailty associations in a 3-year cohort [14], Wang et al. reported that maintaining a low DDS or experiencing a large decline in DDS was associated with higher frailty risk [15], and Weng et al. summarised inverse associations between dietary diversity and frailty in a 2024 systematic review and meta-analysis [16]. The current study adds that even a coarse binary measure of food-group coverage requiring only four yes/no questions captures frailty-relevant dietary information in an Italian geriatric outpatient population where detailed dietary recall may be impractical.

The DDS is conceptually aligned with the minimum dietary diversity (MDD-W) indicator developed by the FAO and validated against micronutrient adequacy in women [24,25,26]. While our DDS captures only four groups rather than the standard ten, its simplicity is precisely its clinical strength. Notably, Otsuka et al. [13] found a similar simplified DDS to predict 4-year cognitive decline in older Japanese adults, and a 2025 systematic review of dietary diversity and healthy ageing concluded that higher dietary diversity is generally associated with favourable ageing-related outcomes, although heterogeneity limits causal inference [27].

### 4.3. The Mediating Role of Nutritional Status

The substantial statistical mediation through MNA (86.2%) is clinically informative but should not be interpreted causally because exposure, mediator, and outcome were measured at the same assessment. A cautious interpretation is that DDS captures broader nutritional status, appetite, meal variety, autonomy in food choice, and protein–energy adequacy. A diet including a greater variety of food groups may be more likely to support adequate intake, while greater sensory variety may also enhance appetite, palatability, and meal engagement in older adults. This interpretation is consistent with ESPEN guidance emphasising routine malnutrition screening and individualised nutritional care in older persons [10], as well as recent evidence linking protein intake patterns with frailty risk [7].

The small residual direct association of DDS with EFS (path c′: β = −0.085, *p* = 0.620) does not exclude micronutrient-related explanations (e.g., vitamin D, B12, folate, zinc) or non-nutrient explanations such as appetite, oral health, cognition, social support, and ability to shop or cook. It does suggest, however, that the MNA-captured nutritional dimension statistically explains most of the observed association in this cohort. Intervention trials are required before concluding that increasing dietary diversity reduces frailty.

### 4.4. Individual Food-Group Findings: Biological Plausibility

The prominence of legumes and eggs in the individual food-group analysis is noteworthy. Legumes, which are rich in plant protein, dietary fibre, isoflavones, and micronutrients (folate, zinc, iron), have shown musculoskeletal and anti-inflammatory associations in observational studies [28]. Eggs provide highly bioavailable complete protein, vitamin D, B12, and choline, all of which are relevant to muscle and neurological function. Their combined classification in our dataset precludes distinguishing their individual contributions. Therefore, the adjusted EFS association (β = −1.339) should be interpreted as a signal for this combined food-group cluster rather than evidence for a specific causal effect of either legumes or eggs alone.

The albumin and CRP associations with fruit and vegetable consumption are consistent with an anti-inflammatory mechanism. Albumin, while primarily a nutritional marker, also reflects systemic inflammation through the negative acute phase response; CRP is a direct inflammatory marker. Habitual consumption of fruits and vegetables—rich in polyphenols, carotenoids, and vitamin C—is associated with lower systemic inflammatory burden [29], and chronic low-grade inflammation (inflammaging) is a recognised driver of frailty [30]. While caution is warranted given the small non-consumer group (*n* = 20), these findings suggest that the frailty benefit of fruits and vegetables may partly operate through an anti-inflammatory pathway distinct from the protein–energy mediating pathway characterising other food groups. However, after Benjamini–Hochberg correction the albumin and CRP associations no longer reached statistical significance (q = 0.075 and q = 0.071, respectively). This putative anti-inflammatory pathway should therefore be interpreted as exploratory and hypothesis-generating rather than established, and warrants confirmation in adequately powered studies with larger numbers of non-consumers.

The dairy association with MNA but not with EFS or SMI in adjusted analyses may reflect the relatively high consumption prevalence (80.9%), which reduces statistical power to detect a between-group difference. Alternatively, it may indicate that dairy intake contributes to nutritional adequacy without providing independent functional information beyond its caloric and protein contribution, an interpretation consistent with the MNA mediation result.

### 4.5. Consistency of Associations Across Sexes

The DDS–EFS association was only statistically significant in women, though the interaction was non-significant and effect sizes were directionally identical in men. The most parsimonious explanation is reduced statistical power in the male subsample (*n* = 83); a conventional power analysis at 80% power applied to the observed male effect size (β = −0.253) would require a substantially larger male sample. The DDS–MNA relationship was significant in both sexes, suggesting equivalent nutritional relevance of dietary diversity across the sex spectrum. Future studies should recruit sex-balanced samples and report sex-stratified analyses by design.

### 4.6. Clinical and Public Health Implications

The DDS has three practical advantages in geriatric care settings: brevity (<2 min), no need for quantitative dietary recall or calculation tools, and integration within existing CGA frameworks alongside EFS and MNA. Our data suggest that a DDS < 3 may serve as a simple clinical prompt to consider a closer nutritional evaluation within the existing CGA pathway. Because the present data are cross-sectional and observational, they do not establish that any specific action triggered by this threshold (e.g., counselling, oral nutritional supplementation, or dietitian referral) improves outcomes; the threshold is offered as a pragmatic screening cue requiring prospective validation. This threshold is supported by the binary sensitivity analysis (DDS = 4 vs. <4: β = −0.793, *p* = 0.015) and by the near-linear gradient of severe frailty across DDS groups (38%, 30%, 15%).

Importantly, dietary diversity is a potentially modifiable and easily communicated dietary target. Brief dietary advice to increase variety, particularly where individual food groups are absent, should be considered a hypothesis-generating clinical strategy rather than evidence from this study of intervention efficacy. If confirmed in prospective studies and trials, the observed effect size (β approximately 0.6–0.8 EFS points per additional food group) could correspond to a clinically meaningful difference in frailty severity in a population where transitions between vulnerability and mild or moderate frailty have major prognostic consequences [31].

### 4.7. Strengths and Limitations

This study has several strengths: a large single-centre geriatric registry (*n* = 1070 registered, 317 in the updated complete-case analytic sample); use of validated, standardised assessment instruments (EFS, MNA, MMSE, ADL, IADL); objective body composition data (DEXA, BIA); formal statistical mediation analysis with bootstrapping; SAGER-compliant sex stratification; regression diagnostic checks; and comprehensive missing data characterisation using the supplied database. The cross-sectional design precludes causal inference; reverse causation cannot be excluded because frailer patients may consume fewer food groups due to functional limitations, dysphagia, dentition problems, appetite loss, cognitive impairment, depressive symptoms, or disease burden. Accordingly, the mediation analysis should be interpreted as statistical mediation only, not as evidence that DDS temporally affects MNA and then frailty. In addition, DDS, MNA, and EFS are conceptually non-independent to some extent: MNA includes nutritional status and dietary/appetite components, and EFS contains a nutrition domain This overlap may inflate statistical mediation estimates. Dietary data were binary and did not capture intake frequency, portion size, preparation method, total energy or protein intake, food quality, appetite, or adherence to dietary patterns such as the Mediterranean diet; DDS therefore serves as a proxy for food-group coverage rather than a direct measure of dietary adequacy. Complete four-item dietary data were available for only 37.7% of the full cohort, introducing potential selection bias; although included and excluded patients were similar on most measured variables, excluded patients used more medications and had a slightly higher diagnostic burden. The complete-case approach may also have introduced bias, particularly because CIRS was missing in 55.5% of the analytic sample, limiting the CIRS-adjusted model to *n* = 141; however, sensitivity adjustment for number of diagnoses and medications in a larger sample yielded similar results. The study was conducted in a single Italian geriatric outpatient centre, limiting external generalisability. Finally, the structured dietary inquiry was not formally validated against a gold-standard food frequency questionnaire in this specific cohort.

## 5. Conclusions

Greater dietary group diversity was dose-responsively associated with lower frailty severity in older adults undergoing geriatric outpatient assessment. This association was largely statistically explained by nutritional status. Legumes/eggs showed the strongest food-specific association with frailty and MNA after FDR correction, whereas fruits/vegetables showed exploratory associations with inflammatory and nutritional biomarkers that did not survive FDR correction. The Dietary Diversity Score is a practical screening tool for identifying patients who may merit fuller nutritional assessment. Longitudinal and interventional studies are required to determine whether increasing dietary diversity can reduce frailty progression.

## Figures and Tables

**Table 1:** Baseline characteristics by Dietary Diversity Score group.

Variable	Low DDS 0–2 (*n* = 50)	Medium DDS 3 (*n* = 101)	High DDS 4 (*n* = 166)	*p* Value
Age, years	83.0 [79–86]	82.0 [79–87]	81.0 [75–85]	0.045 *
Female sex, n (%)	39 (78%)	76 (75%)	119 (72%)	0.622
BMI, kg/m^2^	23.0 [21–28]	23.9 [21–27]	24.9 [22–28]	0.082
No. of medications	8.0 [6–10]	8.0 [6–10]	8.0 [6–11]	0.909
No. of comorbidities	6.0 [5–8]	6.0 [5–8]	6.0 [4–8]	0.419
CIRS severity score	3.0 [2–5]	3.0 [2–5]	3.0 [2–4]	0.469
EFS score	10.0 [7–11]	9.0 [6–11]	8.0 [6–10]	0.008 **
MNA score	17.0 [13–19]	17.0 [15–20]	19.0 [16–20]	<0.001 ***
MMSE score	17.1 [11–23]	20.6 [15–24]	18.1 [14–22]	0.044 *
ADL score	3.0 [2–5]	3.0 [1–5]	4.0 [2–5]	0.109
IADL score	1.0 [0–2]	1.0 [0–2]	1.0 [0–3]	0.217
Albumin, g/dL	3.7 [3–4]	3.7 [3–4]	3.7 [3–4]	0.726
Vitamin D, ng/mL	9.0 [8–14]	10.1 [8–14]	10.9 [8–16]	0.975
CRP, mg/L	0.4 [0–1]	0.3 [0–1]	0.3 [0–1]	0.399
SMI, kg/m^2^	6.5 [6–7]	6.6 [6–7]	6.6 [6–7]	0.261
Handgrip DX, kg	15.5 [12–17]	16.0 [12–20]	16.0 [12–22]	0.150

Values are median [IQR] unless stated. DDS: Dietary Diversity Score; EFS: Edmonton Frail Scale (higher = more frail); MNA: Mini Nutritional Assessment (higher = better nutrition); BMI: body mass index; MMSE: Mini-Mental State Examination; ADL: activities of daily living score (higher = better function); IADL: instrumental activities of daily living score (higher = better function); CRP: C-reactive protein; CIRS: Cumulative Illness Rating Scale; SMI: Skeletal Muscle Index. Kruskal–Wallis or chi-square as appropriate. * *p* < 0.05; ** *p* < 0.01; *** *p* < 0.001.

**Table 2:** Multivariable regression models: association between DDS and EFS.

Model	*n*	**β** DDS (95%CI)	*p*-Value	adj.R^2^
Model 1 Crude	317	−0.660 [−1.024, −0.296]	0.0004 ***	0.036
Model 2 + Age, sex, BMI (main model)	317	−0.608 [−0.976, −0.239]	0.001 **	0.045
Model 3 + CIRS, medications (sensitivity)	141	−0.773 [−1.290, −0.256]	0.004 **	0.069
Model 4 + MNA (mediation test)	313	−0.085 [−0.424, +0.253]	0.620 ns	0.280
Model 5 Ordered logistic (EFS category)	317	OR 0.64 [0.50–0.82]	<0.001 ***	

β: unstandardised coefficient per unit DDS. Models 1–4: linear regression, outcome = EFS continuous. Model 5: ordered logistic regression, outcome = EFS ordinal (5 categories). OR: odds ratio per unit DDS. 95%CI: 95% confidence interval. ns: non-significant. ** *p* < 0.01; *** *p* < 0.001.

## Data Availability

The analysis code (Python scripts reproducing all regression models, the Baron–Kenny bootstrap mediation, and the Benjamini–Hochberg FDR correction) is provided as Supplementary Material and deposited in a public repository to support reproducibility. The de-identified individual-participant dataset contains potentially identifying clinical information from a single-centre geriatric registry and is therefore subject to institutional and ethical restrictions; it is available from the corresponding author upon reasonable request under a formal data-sharing agreement and, where required, additional approval from the competent Ethics Committee.
